# Audiovisual Temporal Perception in Aging: The Role of Multisensory Integration and Age-Related Sensory Loss

**DOI:** 10.3389/fnhum.2018.00192

**Published:** 2018-05-09

**Authors:** Cassandra J. Brooks, Yu Man Chan, Andrew J. Anderson, Allison M. McKendrick

**Affiliations:** Department of Optometry and Vision Sciences, The University of Melbourne, Melbourne, VIC, Australia

**Keywords:** temporal perception, multisensory, audiovisual, integration, audition, vision, aging

## Abstract

Within each sensory modality, age-related deficits in temporal perception contribute to the difficulties older adults experience when performing everyday tasks. Since perceptual experience is inherently multisensory, older adults also face the added challenge of appropriately integrating or segregating the auditory and visual cues present in our dynamic environment into coherent representations of distinct objects. As such, many studies have investigated how older adults perform when integrating temporal information across audition and vision. This review covers both direct judgments about temporal information (the sound-induced flash illusion, temporal order, perceived synchrony, and temporal rate discrimination) and judgments regarding stimuli containing temporal information (the audiovisual bounce effect and speech perception). Although an age-related increase in integration has been demonstrated on a variety of tasks, research specifically investigating the ability of older adults to integrate temporal auditory and visual cues has produced disparate results. In this short review, we explore what factors could underlie these divergent findings. We conclude that both task-specific differences and age-related sensory loss play a role in the reported disparity in age-related effects on the integration of auditory and visual temporal information.

## Introduction

The normal aging process degrades both auditory and visual temporal perception, contributing to the difficulties older adults encounter with everyday tasks. For example, age-related impairments in speech comprehension and driving performance are associated with temporal processing deficits within audition (Gordon-Salant and Fitzgibbons, 1993; Füllgrabe et al., 2014; Babkoff and Fostick, 2017) and vision (Wood, 2002; Conlon and Herkes, 2008; Lacherez et al., 2014), respectively. However, many tasks stimulate both audition and vision. Therefore, this review targets how older adults combine these two sources of temporal information.

Integration binds auditory and visual stimuli into a unified percept, altering their perception relative to how they are perceived in isolation (Ernst and Bülthoff, 2004; Stein et al., 2009). Correctly inferring whether audiovisual integration is appropriate poses a behaviorally relevant challenge. Integration occurs when stimuli seem to share the same origin and one factor influencing this is close temporal correspondence between auditory and visual stimuli, such as similar onset (Lewald and Guski, 2003) and correlated temporal structure (Parise et al., 2012; Denison et al., 2013). Recent reviews indicate that aging increases integration across a range of perceptual domains and behaviors (Freiherr et al., 2013; de Dieuleveult et al., 2017). An age-related increase in integration of congruent auditory and visual cues may be beneficial, for example improving driving performance (Ramkhalawansingh et al., 2016). Conversely, integration of incongruent cues achieves a coherent percept at the cost of veridical perception and so greater integration could hinder everyday function in older adults. Interestingly, review of the literature specifically concerning the integration of temporal information indicates varied age-related effects, with reports of unaltered (e.g., Sommers et al., 2005; McGovern et al., 2014; Brooks et al., 2015) and decreased integration in older adults (e.g., Tye-Murray et al., 2011; Roudaia et al., 2013; Brooks et al., 2015). The perceptual change induced by integration depends on unisensory reliability (the precision of a sensory estimate of a property, as given by the inverse of its variance) and the brain’s tendency to integrate auditory and visual cues (Ernst and Bülthoff, 2004; Odegaard and Shams, 2016). In this minireview, we consider whether age-related sensory loss (which affects unisensory reliability) and task-related differences (which may impact the integration process) explain the diversity of study findings.

## Age-Related Changes in Unisensory Temporal Perception

Physiological aging affects the auditory and visual pathways from the sensory periphery to the cortex, resulting in a decline in many aspects of temporal perception. Within audition, older age reduces sensitivity to temporal fine structure (Grose and Mamo, 2010; Moore et al., 2012; Füllgrabe, 2013; Füllgrabe et al., 2014), amplitude modulation (Takahashi and Bacon, 1992; He et al., 2008; Kumar and Sangamanatha, 2011; Füllgrabe et al., 2014; Wallaert et al., 2016) and frequency modulation (He et al., 2007; Grose and Mamo, 2012; Wallaert et al., 2016). Older adults exhibit impaired auditory gap detection (Snell, 1997; Strouse et al., 1998; Heinrich and Schneider, 2006; Humes et al., 2009; Kumar and Sangamanatha, 2011) and duration discrimination (Fitzgibbons and Gordon-Salant, 1994; Fitzgibbons and Gordon-Salant, 1995; Gordon-Salant and Fitzgibbons, 1999; Kumar and Sangamanatha, 2011), as well as impaired temporal order judgments (Gordon-Salant and Fitzgibbons, 1999; Fitzgibbons et al., 2006; Ulbrich et al., 2009) and temporal sequencing (Trainor and Trehub, 1989).

Within vision, many age-related temporal processing deficits have been documented, including reduced flicker sensitivity (Mayer et al., 1988; Tyler, 1989; Kuyk and Wesson, 1991; Kim and Mayer, 1994), reduced critical flicker frequency (Misiak, 1947, 1951; Coppinger, 1955; McFarland et al., 1958; Lachenmayr et al., 1994) and some impairments in motion perception (Trick and Silverman, 1991; Tran et al., 1998; Habak and Faubert, 2000; Snowden and Kavanagh, 2006; Bennett et al., 2007; Billino et al., 2008). Similar to deficits in auditory processing, there is an age-related decline in visual gap detection (Humes et al., 2009) and temporal order judgments (Ulbrich et al., 2009; Busey et al., 2010; de Boer-Schellekens and Vroomen, 2014).

Furthermore, sensory decline is typically uneven across vision and audition, though the more adversely affected sensory modality varies between tasks (Čeponienė et al., 2008; Guerreiro and Van Gerven, 2011; Lustig and Meck, 2011; Cliff et al., 2013; Diaconescu et al., 2013; Guerreiro et al., 2013). For example, older age impairs visual duration judgments more than auditory ones (Lustig and Meck, 2011). In contrast, auditory but not visual temporal rate discriminability is poorer in older adults (Brooks et al., 2015). Consequently, for a given audiovisual task, auditory and visual cues are not expected to be equally degraded by age-related sensory changes.

## Age-Related Changes in Audiovisual Temporal Perception

Studies have investigated age-related changes to various measures of audiovisual temporal perception presumed to reflect multisensory integration, with disparate results for transient stimuli like flashes and beeps. Older adults are more susceptible to the sound-induced flash illusion (Setti et al., 2011a; DeLoss et al., 2013; McGovern et al., 2014). In this illusory fission effect, the integration of two beeps and a solitary flash results in the perception of two flashes (Shams et al., 2000). Older adults also exhibit an enhanced temporal ventriloquist effect (de Boer-Schellekens and Vroomen, 2014), in which the presentation of a click before and after a sequence of two flashes increases visual temporal order sensitivity (Morein-Zamir et al., 2003). Conversely, the integration of two flashes with a single beep results in the perception of a single flash in an illusory fusion effect (Andersen et al., 2004) that does not change in aging (McGovern et al., 2014). In the audiovisual bounce effect, an auditory beep influences a visual spatiotemporal illusion, in which two disks moving toward each other appear either to stream past one another, or less frequently, to bounce (Sekuler and Sekuler, 1999). The beep, sounding as the disks overlap, causes an increase in the frequency of perceived bouncing (Sekuler et al., 1997). This effect is reduced in older adults, suggestive of decreased integration (Roudaia et al., 2013).

Age-related effects on the integration of temporally modulated stimuli depend on audiovisual congruency. Partial integration of non-identical auditory and visual temporal rates distorts perceived rate such that the auditory or visual rate subjectively equivalent to a reference is non-veridical (Roach et al., 2006). This effect is equivalent in younger and older adults (Brooks et al., 2015). However, integration of identical auditory and visual temporal rates improves temporal rate discrimination in a statistically optimal fashion in younger (Koene et al., 2007; Brooks et al., 2015) but not older adults, who fail to show the √ 2 improvement predicted by maximum likelihood estimation (Brooks et al., 2015).

Research on audiovisual speech perception in aging is also relevant to this review, since the temporal relationships between auditory and visual speech cues (Chandrasekaran et al., 2009)facilitate both auditory speech detection (Grant and Seitz, 2000) and recognition (ten Oever et al., 2013; Jaekl et al., 2015). Visual facilitation of auditory speech detection is reduced in older adults (Tye-Murray et al., 2011). The effect of aging on audiovisual gains in speech recognition is more complex, with no age-related changes evident when auditory and visual speech cues are clear and congruent (Ballingham and Cienkowski, 2004; Sommers et al., 2005; Spehar et al., 2008; Gordon and Allen, 2009; Legault et al., 2010; Tye-Murray et al., 2010; Winneke and Phillips, 2011; Huyse et al., 2014; Smayda et al., 2016; Sommers and Phelps, 2016). However, audiovisual gains are reduced in older adults when auditory and visual speech cues are degraded (Tye-Murray et al., 2008, 2010, 2011; Gordon and Allen, 2009; Huyse et al., 2014; Stevenson et al., 2015) or asynchronous (Gordon-Salant et al., 2017).

In the McGurk effect, integration of incongruent auditory and visual speech results in a fused percept that matches neither cue (McGurk and Macdonald, 1976). Most studies report a similar proportion of fused responses in younger and older adult groups for syllables (Cienkowski and Carney, 2002; Ballingham and Cienkowski, 2004; Huyse et al., 2014; Stothart and Kazanina, 2016). Discrepant findings reported in the literature likely reflect differences in study design, with an age-related increase in fused responses found when speech stimuli were words (Setti et al., 2013) and an age-related decrease in fused responses found when auditory syllables were presented in modulated noise (Huyse et al., 2014). Comparison between studies using different experimental stimuli is also complicated by known variability in the McGurk effect with different speakers (Jiang and Bernstein, 2011; Mallick et al., 2015) and different auditory and visual syllable pairings (Jiang and Bernstein, 2011).

Altogether, research indicates an age-related increase in the integration of temporally offset auditory and visual events. Older adults perceive the sound-induced flash illusion at larger temporal offsets between auditory and visual stimuli than younger adults (Setti et al., 2011a; McGovern et al., 2014). When judging synchrony, older adults perceive temporally offset auditory and visual events as simultaneous over a broader (Hay-McCutcheon et al., 2009; Noel et al., 2016), narrower (Alm and Behne, 2013) or similar (Baskent and Bazo, 2011; Bedard and Barnett-Cowan, 2016) temporal window compared to younger adults. However, the temporal window of perceived synchrony is broader in older adults when individual differences in stimulus detectability and response criterion are normalized, indicative of enhanced integration (Chan et al., 2014). Similarly, in judgments of temporal order, older adults need a larger temporal gap between an auditory and visual stimulus to distinguish which appeared first (Virsu et al., 2003; Setti et al., 2011b; de Boer-Schellekens and Vroomen, 2014; Bedard and Barnett-Cowan, 2016), but one study found no age-related differences in audiovisual temporal order judgments (Fiacconi et al., 2013). All of these temporal order studies used stimuli that were not individually scaled to detectability, and Fiacconi et al tested observers of at least 70 years of age, which is older than the inclusion criteria of the other above cited studies (at least 60 years).

In summary, integration of temporal auditory and visual information may be increased, decreased or unchanged by physiological aging (see **Tables 1**, **2**). This suggests that age-related effects on integration may be task-specific, as discussed in the next section.

**Table 1:** Age-related changes in the perception of audiovisual temporal information consistent with decreased, unchanged, or increased integration in older relative to younger adults.

Audiovisual task	Age-related changes in multisensory integration:		
	
	*Decreased*	*Unchanged*	*Increased*
Sound-induced flash fission	–	–	Setti et al., 2011a; DeLoss et al., 2013; McGovern et al., 2014
Sound-induced flash fusion	–	McGovern et al., 2014	–
Temporal ventriloquist effect	–	–	de Boer-Schellekens and Vroomen, 2014
Audiovisual bounce effect	Roudaia et al., 2013	–	–
Identical temporal rates	Brooks et al., 2015	–	–
Non-identical temporal rates	–	Brooks et al., 2015	–
Speech detection	Tye-Murray et al., 2011	–	–
Speech recognition	Tye-Murray et al., 2008, 2010, 2011; Gordon and Allen, 2009; Huyse et al., 2014; Stevenson et al., 2015; Gordon-Salant et al., 2017	Ballingham and Cienkowski, 2004; Sommers et al., 2005; Spehar et al., 2008; Gordon and Allen, 2009; Legault et al., 2010; Tye-Murray et al., 2010; Winneke and Phillips, 2011; Huyse et al., 2014; Smayda et al., 2016; Sommers and Phelps, 2016	–
McGurk effect (fused responses)	Huyse et al., 2014	Cienkowski and Carney, 2002; Ballingham and Cienkowski, 2004; Huyse et al., 2014; Stothart and Kazanina, 2016	Setti et al., 2013


*See text for discussion of how closely changes in perceptual measures reflect the underlying ability to integrate.*

**Table 2:** Age-related changes in the temporal binding window of auditory and visual events, indicating whether older adults were found to have narrower, unchanged, or wider temporal binding windows relative to younger adults.

Audiovisual task	Age-related changes in the temporal binding window:		
	
	*Narrower*	*Unchanged*	*Wider*
Temporal order judgments	–	Fiacconi et al., 2013	Virsu et al., 2003; Setti et al., 2011b; de Boer-Schellekens and Vroomen, 2014; Bedard and Barnett-Cowan, 2016
Synchrony judgments	Alm and Behne, 2013	Baskent and Bazo, 2011; Bedard and Barnett-Cowan, 2016	Hay-McCutcheon et al., 2009; Chan et al., 2014; Noel et al., 2016


## Task-Specific Integration Processes

An outstanding question is how closely audiovisual integration of temporal information is linked across different tasks. On the one hand, performance across different temporal audiovisual tasks is often correlated within individuals (Tremblay et al., 2007; Stevenson et al., 2012; Stevenson and Wallace, 2013), suggesting that different tasks can index common processes. Accordingly, older adults exhibit both increased susceptibility to the sound induced flash illusion (Setti et al., 2011a; DeLoss et al., 2013; McGovern et al., 2014) and broader temporal binding windows (Chan et al., 2014; Noel et al., 2016), consistent with the correlation between performance on these tasks in younger adults (Stevenson et al., 2012). However, within-subject correlations in younger adults have a limited potential to connect age-related effects on integration across different tasks. Correlations between tasks may reflect unisensory reliability, rather than a shared tendency to integrate, as both factors contribute to observed audiovisual interactions (Odegaard and Shams, 2016). Preliminary evidence suggests that the brain’s tendency to integrate auditory and visual cues is task-specific, though it remains unclear how tightly integration tendency is linked across tasks within the same perceptual domain (Odegaard and Shams, 2016). Furthermore, while low level stimulus characteristics such as temporal correspondence and unisensory reliability modulate the strength of audiovisual interactions, the integration process is also influenced by contextually-driven factors such as task goal, attention, and learned audiovisual associations (for review see van Atteveldt et al., 2014; Tye-Murray et al., 2016).

Different tasks likely index different perceptual processes. For example, research suggests distinctions between the perception of audiovisual temporal rate and relative timing (Fujisaki and Nishida, 2005). Moreover, a complex task like audiovisual speech perception is influenced by higher level factors, such as semantic congruence and linguistic meaning, not just lower level stimulus characteristics like temporal structure (Eskelund et al., 2011; Lee and Noppeney, 2011; Stekelenburg and Vroomen, 2012; ten Oever et al., 2013; Stevenson et al., 2014). In older adults, audiovisual gains in speech recognition decrease with task complexity, such as lexical difficulty (Dey and Sommers, 2015) or whole-word compared to phoneme recognition (Stevenson et al., 2015).

Additionally, audiovisual temporal tasks may not index the same perceptual processes even with comparable stimuli, such as audiovisual speech detection and recognition(Eskelund et al., 2011) and audiovisual temporal order and synchrony judgments (Love et al., 2013). In fact, estimates of the temporal binding window from temporal order and synchrony tasks are not correlated in older adults (Bedard and Barnett-Cowan, 2016). The McGurk effect is experienced at temporal offsets between auditory and visual syllables where observers are aware of the asynchrony (Soto-Faraco and Alsius, 2009), in line with differences in the neural substrate processing asynchrony and perceptual fusion for audiovisual speech (Stevenson et al., 2011). Furthermore, evidence points toward distinct cortical mechanisms of illusory flash fusion and fission (Mishra et al., 2007, 2008) that may be differentially vulnerable to age-related effects given differences in older adult susceptibility to each illusion type (McGovern et al., 2014). Likewise, older adults exhibit impaired integration of identical but not conflicting auditory and visual temporal rates, suggesting the presence of separate integration mechanisms that differ in their susceptibility to age-related decline (Brooks et al., 2015).

Lastly, there are concerns regarding the adequacy of some study measures as indices of integration. Though some argue that decisional processes drive the audiovisual bounce effect (Grove et al., 2016; Zeljko and Grove, 2016), most studies indicate that the effect is perceptual (Watanabe and Shimojo, 2001; Sanabria et al., 2004; Dufour et al., 2008; Meyerhoff and Scholl, 2018). Multiple studies suggest that the McGurk effect is an unreliable measure of integration, due to substantial individual variability (Mallick et al., 2015), underestimation of visual modulation of the auditory cue (Brancazio and Miller, 2005; Tiippana, 2014) and the contribution of individual differences in sensitivity to incongruity and lipreading skill to illusion susceptibility (Strand et al., 2014). As for audiovisual gains in speech recognition, recent analysis argues improved performance does not result from integration and that different integration measures indicate opposing age-related effects (Tye-Murray et al., 2016). Greater understanding of the relationships between audiovisual tasks and how closely perceptual changes reflect integration is needed to clarify current research.

## The Role of Age-Related Changes in Unisensory Processing

Age-related unisensory changes are a potential confounding factor in the interpretation of age-related changes in audiovisual perception. According to the principle of inverse effectiveness, if auditory and visual cues are less salient, there will be a greater proportionate change in neural responses upon their integration (Meredith and Stein, 1983). Since this principle can also apply to perception (Stein et al., 1988), apparently enhanced integration in older age may reflect reduced stimulus saliency due to age-related sensory loss (Mozolic et al., 2012; Freiherr et al., 2013). However, a relationship between sensory reliability and integration does not necessarily mean that age-related sensory loss will explain age-related differences. While lower stimulus intensities broaden temporal binding windows in younger adults (Krueger Fister et al., 2016), older adults demonstrated broader temporal binding windows than younger adults when stimulus intensity was scaled to individual detection thresholds, indicating the effect was independent of age-related declines in sensory sensitivity (Chan et al., 2014). Furthermore, counter to the predictions of inverse effectiveness, there is an age-related increase in audiovisual enhancement of word recognition at intermediate auditory signal to noise ratios (Stevenson et al., 2015) and an age-related decrease in audiovisual enhancement of degraded sentences (Tye-Murray et al., 2010). This is consistent with evidence that audiovisual gains in speech recognition are greatest for intermediate auditory clarity (Ross et al., 2007; cf. Stevenson et al., 2015).

The reliability of auditory relative to visual sensory estimates of a property (where reliability is defined as the estimate’s precision, as given by the inverse of its variance) also influences audiovisual interactions (Ernst and Bülthoff, 2004). In contrast to the historical view that audition dominates temporal perception due to its superior temporal resolution (Welch and Warren, 1980), current theory holds that the brain weights a pair of sensory cues according to their relative reliability to derive the most precise multisensory representation possible from available information (Ernst and Bülthoff, 2004; Witten and Knudsen, 2005; Fetsch et al., 2013). Unisensory reliability plays a role in susceptibility to audiovisual illusions, in which information from one sensory modality modulates perception in another (e.g., Sekiyama and Tohkura, 1991; Andersen et al., 2005; Roach et al., 2006; Kumpik et al., 2014; Strand et al., 2014). Consequently, uneven age-related sensory decline could alter the relative contribution of audition and vision to the audiovisual percept, resulting in an apparent change in illusion susceptibility. Age-related shifts in the weighting of auditory and visual cues have been documented for audiovisual speech. When studies employed audiovisual speech cues that gave rise to age-related differences in unisensory performance, older adults gave more weight than younger adults to auditory (Huyse et al., 2014) or visual information (Cienkowski and Carney, 2002; Sekiyama et al., 2014; Festa et al., 2017), in accordance with the more reliable sensory modality. However, it is possible to probe age-related differences in integration capacity by individually balancing the relative reliability of auditory and visual cues, as was shown for temporal rate perception (Brooks et al., 2015).

Unfortunately, studies of audiovisual integration in older adults have not employed a consistent definition of what constitutes normal hearing and vision, and may not screen both sensory modalities for age-abnormal changes. Even so, screening measures commonly used in older adults such as audiometric thresholds and visual acuity are poor indicators of other aspects of auditory (e.g., Gordon-Salant and Fitzgibbons, 1999; Plack et al., 2014) or visual perception (e.g., Haegerstrom-Portnoy et al., 1999), respectively. The issue is further complicated by the often indistinct boundary between age-related and pathological changes (Owsley, 2011). Older age increases the prevalence of ocular diseases such as glaucoma (Quigley and Broman, 2006) and age-related macular degeneration (Friedman et al., 2004) that can further impair temporal perception even in early stages (e.g., Ansari et al., 2002; Phipps et al., 2004; Spry et al., 2005; Dimitrov et al., 2011; Gin et al., 2011). Age-related sensorineural hearing loss contributes to impaired auditory temporal perception independently from physiological aging (Gallun et al., 2014) and for stimuli at low frequencies despite normal audiometric thresholds (Feng et al., 2010). Age-related high frequency hearing loss also increases the intrinsic functional connectivity between auditory and visual cortical regions, which may increase audiovisual interactions in older adults (Puschmann and Thiel, 2017). Before drawing conclusions on how integration may change with age, studies should carefully account for age-related changes in both auditory and visual saliency on an individual basis (e.g., Chan et al., 2014; Brooks et al., 2015).

## Conclusion

Audiovisual integration may not necessarily provide older adults with an effective compensatory mechanism for age-related unisensory decline in temporal information. While older adults often benefit from the provision of complimentary auditory and visual cues, it can be to a reduced extent compared to younger adults. Older adults sometimes demonstrate an increased tendency to synthesize conflicting temporal auditory and visual cues into a unified percept but this is not a universal finding. Further research elucidating how closely integration is linked across different audiovisual tasks is needed to clarify this pattern of results. Currently, it remains unclear to what extent age-related changes in audiovisual temporal perception reflect true changes in integration rather than sequential effects of age-related sensory loss. Future research should account for individual differences in unisensory reliability to distinguish age-related sensory loss from age-related effects on integration.

## Author Contributions

CB: conception and planning of review and wrote the manuscript. YC: planning of review and contributed to the writing of Section “Age-Related Changes in Audiovisual Temporal Perception.” AM and AA: conception of review and revision of the manuscript. All authors approved the manuscript.

## Conflict of Interest Statement

The authors declare that the research was conducted in the absence of any commercial or financial relationships that could be construed as a potential conflict of interest.
